# Mitochondrial Function Is Required for Secretion of DAF-28/Insulin in *C. elegans*

**DOI:** 10.1371/journal.pone.0014507

**Published:** 2011-01-17

**Authors:** Ola Billing, Gautam Kao, Peter Naredi

**Affiliations:** Department of Surgery and Perioperative Sciences, Umeå University, Umeå, Sweden; National Institute on Aging, United States of America

## Abstract

While insulin signaling has been extensively studied in *Caenorhabditis elegans* in the context of ageing and stress response, less is known about the factors underlying the secretion of insulin ligands upstream of the insulin receptor. Activation of the receptor governs the decision whether to progress through the reproductive lifecycle or to arrest growth and enter hibernation. We find that animals with reduced levels of the mitochondrial outer membrane translocase homologue TOMM-40 arrest growth as larvae and have decreased insulin signaling strength. TOMM-40 acts as a mitochondrial translocase in *C. elegans* and in its absence animals fail to import a mitochondrial protein reporter across the mitochondrial membrane(s). Inactivation of TOMM-40 evokes the mitochondrial unfolded protein response and causes a collapse of the proton gradient across the inner mitochondrial membrane. Consequently these broadly dysfunctional mitochondria render an inability to couple food abundance to secretion of DAF-28/insulin. The secretion defect is not general in nature since two other neuropeptides, ANF::GFP and INS-22::VENUS, are secreted normally. RNAi against two other putative members of the TOMM complex give similar phenotypes, implying that DAF-28 secretion is sensitive to mitochondrial dysfunction in general. We conclude that mitochondrial function is required for *C. elegans* to secrete DAF-28/insulin when food is abundant. This modulation of secretion likely represents an additional level of control over DAF-28/insulin function.

## Introduction

All metazoans need to be able to adapt to changing food levels in the environment. Absence or presence of food needs to be sensed and conveyed to elicit appropriate behavioral and developmental responses. Failure to do so results in an inability to adapt to changes in nutritional status and ultimately in an evolutionary disadvantage. Through the development of insulin signaling, metazoans have adapted to a life with changing food supplies. In insulin-secreting cells, increasing levels of energy are converted into various stimulatory signals that trigger fusion of insulin-containing vesicles to the plasma membrane, consequently releasing insulin. The binding of insulin to its receptor activates a kinase cascade, which in the end prevents a FOXO-type transcription factor from entering the nucleus. When less insulin is secreted, the transcription factor is unphosphorylated and free to enter the nucleus, where it activates starvation-specific genes. This rationale of insulin signaling is conserved from nematodes to humans.

In *Caenorhabditis elegans*, the insulin signaling pathway plays an important role in regulating larval and dauer diapause. In the presence of food, worms progress through four larval stages (L1–L4) to reach adulthood. In absence of food, worms can enter hibernation at three distinct stages during their development. If worms hatch in the absence of food they arrest growth reversibly as mid-stage L1 larvae [1], [2]. Towards the end of this stage a second decision is made, whether or not to become dauers instead of L3 larvae. Aside from food levels, this decision also depends on temperature and population density [3]. There is also a reproductive diapause at the adult stage, in which hermaphrodites in the absence of food arrest embryogenesis in their gonads and form no more than two embryos [4]. The L1 and dauer diapause stages can be entered inappropriately in various mutants that are compromised for insulin signaling. For instance, a severe mutation in the DAF-2/insulin receptor causes an L1 arrest, similar to that of wild type animals that hatch in the absence of food. Less severe perturbations to the insulin pathway can cause increased entry into the dauer stage or into the adult diapause. Conversely, “over activation” of the insulin pathway by mutations in *daf-16/FOXO* results in an inability to enter diapause when food is limiting [3].

The *C. elegans* genome encodes forty insulin-like peptides (ILPs) [5], [6]. One of them, DAF-28, has some hallmarks of a mammalian-type insulin, regulating metabolic homeostasis. *daf-28* responds to changes in food levels and plays an important role in coordinating overall metabolic and anatomical responses to changes in nutritional status [7]. High levels of DAF-28 protein promote reproductive growth and prevent hibernation in the dauer stage during nutritionally replete conditions. Its level of transcription is high when food levels are high [7]. At the level of secretion, DAF-28 can be studied using a DAF-28::GFP reporter in intact worms. The use of this reporter has shown that its secretion is sensitive to nutritional status, so that less DAF-28::GFP is secreted in worms that are starved [8], which indicates function as a metabolic insulin.

Insulin secretion has been most extensively studied in mammals, where it has been shown to be influenced by mitochondria in several ways. The major stimulatory influence is via mitochondrial production of ATP from ADP. An increased ATP/ADP ratio results in closure of ATP-gated K^+^ channels, which is a key step in insulin secretion. Mitochondria are also thought to stimulate insulin secretion by means of secretion-stimulatory intermediates that are generated by its intermediary metabolism. These include pyruvate, glutamate and malonyl-CoA [9]. In addition, mitochondria-derived reactive oxygen species were recently shown to be obligatory signals for glucose stimulated insulin secretion in perfused rat islets [10]. At a pathological level, mitochondrial failure, by means of mutations in mitochondrial DNA, can cause mitochondrial diabetes in humans and mice [11], [12]. The importance of mitochondria in insulin secretion has been further highlighted in several studies, showing that glucose-stimulated insulin secretion is reduced or abolished when mitochondria are dysfunctional [12], [13].

Although separated from the cytosol, mitochondria are largely dependent on communication with the nucleus. The mitochondrial genome encodes only a minority of the approximately 800–1500 proteins, depending on species that function in the organelle. The vast majority of the mitochondrial proteins are encoded by the nuclear genome and are synthesized in the cytosol. Thus, they need to be imported from the cytosol across the mitochondrial membrane(s). All preproteins that are destined to enter the mitochondrion must first pass through the gated protein pore of the TOM complex (translocase of the outer mitochondrial membrane). The TOM40 protein constitutes the actual hydrophilic pore through which all preproteins are imported [14], [15]. Some subunits of the complex, such as TOM20 and TOM22, are receptors that recognize different subgroups of mitochondria-destined preproteins, while smaller subunits like TOM7 contribute to the stability of the complex [16]. In *C. elegans*, there are few reports describing functions of putative TOM-complex subunit homologues. RNAi against *tomm-7* was shown to affect mitochondrial morphology, TOMM-20 has been shown to localize to mitochondrial membranes [17] and *tomm-40* was identified in an RNAi screen for genes affecting mitochondrial morphology [18].

To find new regulators of *C. elegans* insulin signaling, we performed an RNAi screen for genes which, when inactivated, caused larval arrest. RNAi against *tomm-40* caused a larval growth arrest, with reduced signaling through the insulin pathway in spite of normal ability to feed. We show that a reduced level of TOMM-40 resulted in a defect in mitochondrial protein import, and consequently in severe mitochondrial dysfunction. These functionally compromised mitochondria failed to promote secretion of DAF-28/insulin. This was also seen upon RNAi against two other members of the TOMM complex. However, the secretion defect was not general in nature, since two other dense core vesicle residing proteins, ANF::GFP and INS-22::VENUS were secreted normally. TOMM-40 likely acted in neurons to influence insulin signaling and overexpression of DAF-28/insulin rescued the insulin signaling defect caused by depletion of TOMM-40. Since DAF-28/insulin secretion in this way is sensitive to TOMM complex dysfunction, we conclude that mitochondria in general and the TOMM complex in particular are required to couple food signals to secretion of DAF-28/insulin.

## Results

### TOMM-40 is a broadly expressed protein that localizes to mitochondria

**Figure 1:**
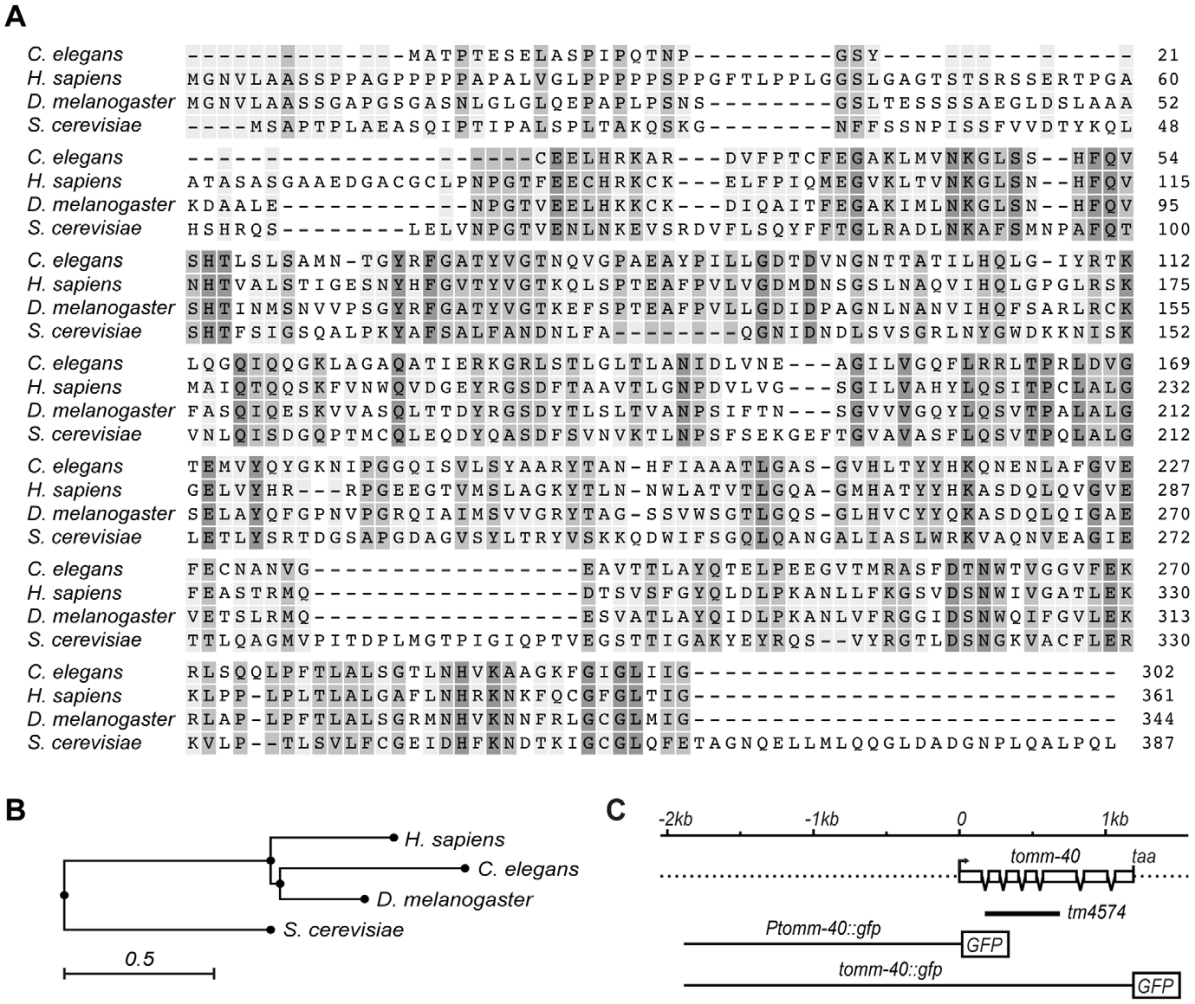
TOMM-40 is the *C. elegans* homologue of TOM40. (**A**) Amino acid sequence comparison of TOMM-40 homologues from *C. elegans, H. sapiens, D. melanogaster* and *S. cerevisiae*. Dark gray corresponds to 100% interspecies conservation, medium gray to 75% and light gray to 50%. (**B**) Phylogenetic tree analysis of TOMM-40 homologues. The scale indicates sequence difference, where 0.5 is 50% difference in residues. The tree was constructed using the neighbor-joining algorithm. (**C**) (From top to bottom) Scale bar, followed by the intron/exon structure of *tomm-40*. Boxes represent exons. The first methionine is indicated by an arrow. (Below) The tm4574 mutation deletes exon two, three and four and part of exon five and instead inserts 28 base pairs in the open reading frame of *tomm-40* are indicated with a black bar. (Below) The P*_tomm-40_::gfp* (pVB488OB) and the P*_tomm-40_::tomm-40::gfp* (pVB518OB) constructs used in this study are shown.

Based on sequence similarity to TOM40 homologues in *Homo sapiens*, *Drosophila melanogaster* and *Saccharomyces cerevisiae*, *C. elegans* TOMM-40 is predicted to be a mitochondrial translocase subunit [Figure 1A, B and [19]]. It shares 49% and 42% protein sequence identity with *D. melanogaster* and *H. sapiens* TOM40 respectively. The *C. elegans tomm-40* gene (cosmid name: C18E9.6) contains seven exons, encoding a 301 amino acid protein. The cDNA was completely sequenced and shown to possess the predicted splicing pattern.

**Figure 2:**
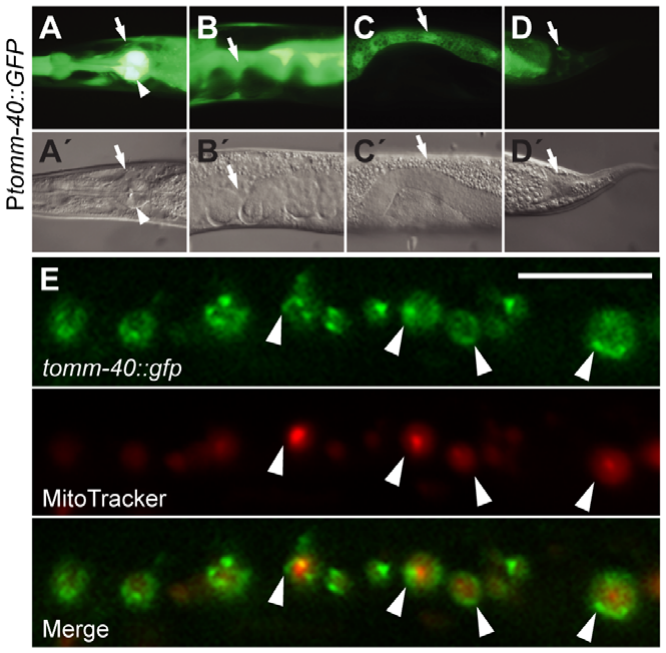
TOMM-40 is a broadly expressed mitochondrial membrane protein. (**A-D**) Fluorescence images showing GFP expression from the P*_tomm-40_::gfp* construct and (**A'-D'**) the corresponding Nomarski images. GFP expression is indicated in: (**A**) The nerve ring (arrow) and pharyngeal muscle (arrowhead), (**B**) gonadal sheath, (**C**) the intestine, (**D**) tail hypodermis. (**E**) Confocal imaging of a body wall muscle cell expressing TOMM-40:GFP and stained with mitotracker dye. TOMM-40::GFP expressed from pVB518OB localized in rings that enclosed mitochondrial matrix associated red MitoTracker dye. Arrowheads indicate TOMM-40::GFP localized to mitochondrial membranes. The scale bar is 10 µm.

*tomm-40* was expressed at high levels in pharyngeal muscles, the nerve ring, the intestine, gonadal sheath and in tail hypodermis (Figure 2A–D), as visualized by GFP expression from the transcriptional fusion construct P*_tomm-40_::gfp* (Figure 1C). To study its subcellular localization, we made a full-length C-terminal fusion of GFP to the *tomm-40* cDNA under its own promoter. The TOMM-40::GFP fusion protein was also expressed in a ubiquitous pattern, similar to that seen with the transcriptional gfp reporter. At a sub-cellular level TOMM-40::GFP localized to mitochondrial membranes, as shown by localization around red MitoTracker staining, which accumulates in the mitochondrial matrix (Figure 2E, Figure S1). No localization of the protein was seen outside mitochondria. This type of localization pattern has been reported for other worm mitochondrial membrane proteins including another member of the TOMM complex [17], [20] and it was consistent with the expected role of TOMM-40.

### TOMM-40 is a positive regulator of growth

**Figure 3:**
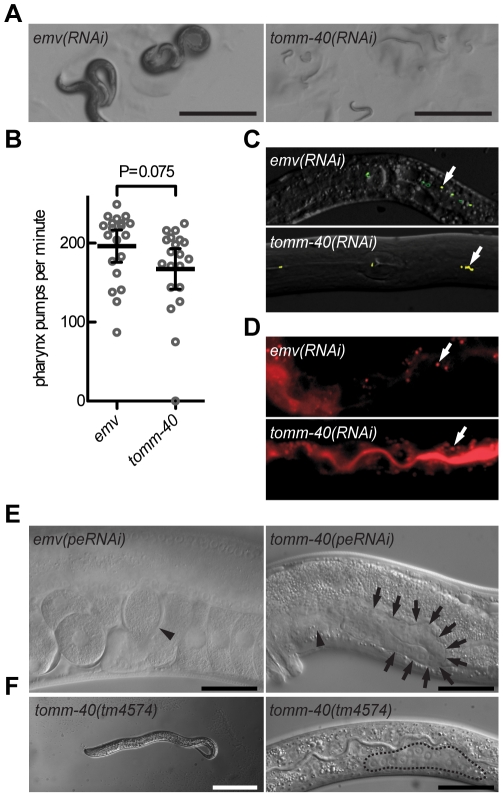
TOMM-40 promotes growth. (**A**) Animals fed with bacteria containing empty vector (emv), were adults 72 hours after hatching while *tomm-40(RNAi)* caused growth arrests, at various stages, in the same time period. Scale bars are 1 mm. (**B–D**) Feeding analysis of worms arrested as L1s either by *tomm-40(RNAi)* or by hatching in the absence of food. Control animals were fed with emv-containing bacteria 20 minutes prior to analysis. (**B**) Pharyngeal pumping rates. Circles denote data from individual worms, horizontal lines represent mean values, and bars show a confidence interval of 95%. (**C**) Micrographs of arrested worms incubated on lawns of bacterium-sized fluorescent beads. Arrows indicate beads localized in the intestinal lumen. (**D**) FM4-64 stainings. Arrows denote endocytic vesicles containing the dye. (**E**) Nomarski pictures of the gonad and the uterus in a fertile adult treated with emv post embryonic RNAi [*emv(peRNAi)*] and a sterile *tomm-40(peRNAi)* treated adult. In the left panel, the arrowhead indicates an oocyte undergoing ovulation. In the right panel the arrowhead indicates the absence of fertilized embryos in the uterus. Arrows indicate a mis- migrated gonad arm Scale bars are 25 µm. (**F**) A *tomm-40(tm4574)* animal arrested permanently as an L2 larva. The dashed line outlines the gonad. Scale bars are 100 µm (white) and 25 µm (black).

Reducing TOMM-40 levels, by exposing 4^th^ stage larvae to feeding RNAi, causes a growth retardation in their progeny with most animals arresting as embryos or between the 1^st^ and the 3^rd^ larval stages (Figure 3A). As an index of the growth defect, the number of larvae in the 1^st^ larval stage (L1) was counted 24 hours after removing their mothers in a feeding RNAi assay. While only 0.4% (n = 223) of animals fed with *emv(RNAi)* bacteria were in stage L1, 60% (n = 194) of *tomm-40(RNAi)* animals were in this stage. Later, most of the larvae appeared to arrest permanently at the L2 or L3 stage although many still remained in the L1 stage. Using high power microscopy, we could not detect any catastrophic developmental defects that might cause the larval arrest.

Since *tomm-40* was ubiquitously expressed, we asked if the observed growth defect was due to any pharyngeal or intestinal defects, which would cause feeding defects. We did so in three ways. First, the rate of food ingestion was assessed by measuring pharyngeal pumping rates. We compared animals arrested as L1s by *tomm-40(RNAi)* to wild type age-matched larvae. Pharyngeal pumping rates in *tomm-40(RNAi)* larvae were not significantly different from wild type larvae (Figure 3B). Second, we found that the feeding motor program was normal in arrested animals, since the ability to ingest bacterium-sized beads into the gut lumen was unaffected (Figure 3C). Third, we examined endocytosis of FM4-64, a dye that is taken up by endocytic vesicles in the intestine. *tomm-40(RNAi)* arrested animals appeared similar to *emv(RNAi)* controls in their ability to form FM4-64-containing endocytic vesicles in intestinal cells (Figure 3D). Taken together, the feeding analyses showed no significant difference between *tomm-40(RNAi)* and *emv(RNAi)* animals. Therefore we conclude that the primary cause of the severe growth arrest was not due to a defect in their ability to feed.

TOMM-40 levels were reduced to a lesser extent by a weaker form of feeding RNAi termed “postembryonic RNAi” (peRNAi). This procedure allowed TOMM-40 to function normally during germline and embryonic development and reduced protein levels later, during larval and adult stages. peRNAi against *tomm-40* allowed worms to develop to adulthood instead of causing larval arrest, although most of them were sterile. 34 of the 38 *tomm-40(peRNAi)* adults produced no progeny while the rest produced less then 15 progeny each, which is 5% of a wild type brood size. Some of these sterile animals produced oocytes and sperm but lacked fertilized embryos (Figure 3E). The more severely affected worms displayed abnormal gonad migration and lacked oocytes, but were otherwise wild type in appearance.

A deletion mutation in the gene, *tomm-40(tm4574)*, was obtained from Dr. Shohei Mitani and the National Bioresource Project, Tokyo. The mutation deletes several exons (Figure 1C) and is a likely to be a null mutation. We examined the mutant to test whether the larval arrest phenotype seen in *tomm-40(RNAi)* animals was also seen in *tm4574* mutants. The strain was outcrossed five times and maintained as a heterozygote over a balancer chromosome since *tomm-40(tm4574)* homozygotes arrested as larvae. Using the number of somatic gonadal cells as a criterion for age, 77% of the arrested larvae were found to be L2 larvae, 16% were L3 larvae and 7% were L1 larvae (n = 43) (Figure 3F). All the progeny from *tomm-40(tm4574)/balancer* mothers that grew to adulthood, and were not *balancer/balancer* homozygotes, were heterozygous for *tm4574* (n = 106). No embryonic lethality was detected among the progeny of the heterozygous strain and the number of arrested larvae segregating from a heterozygote was equal to the number of balancer homozygotes. All arrested larvae that were genotyped by PCR were homozygous for the deletion allele. Taking these observations together, we conclude that *tomm-40(tm4574)* homozygotes display a strict larval arrest. This observation is consistent with the larval arrest phenotype seen in *tomm-40(RNAi)* animals. Examination of the mutant larvae by high power microscopy showed no visible morphological abnormalities, except for a pale appearance. They but had normal locomotion for up to 7 days after which most of them died.

In summary, TOMM-40 was required continuously for growth during embryogenesis and during all postembryonic stages. TOMM-40 was also needed for germline and somatic gonad development.

### Mitochondrial function is diminished in animals with reduced levels of TOMM-40

**Figure 4:**
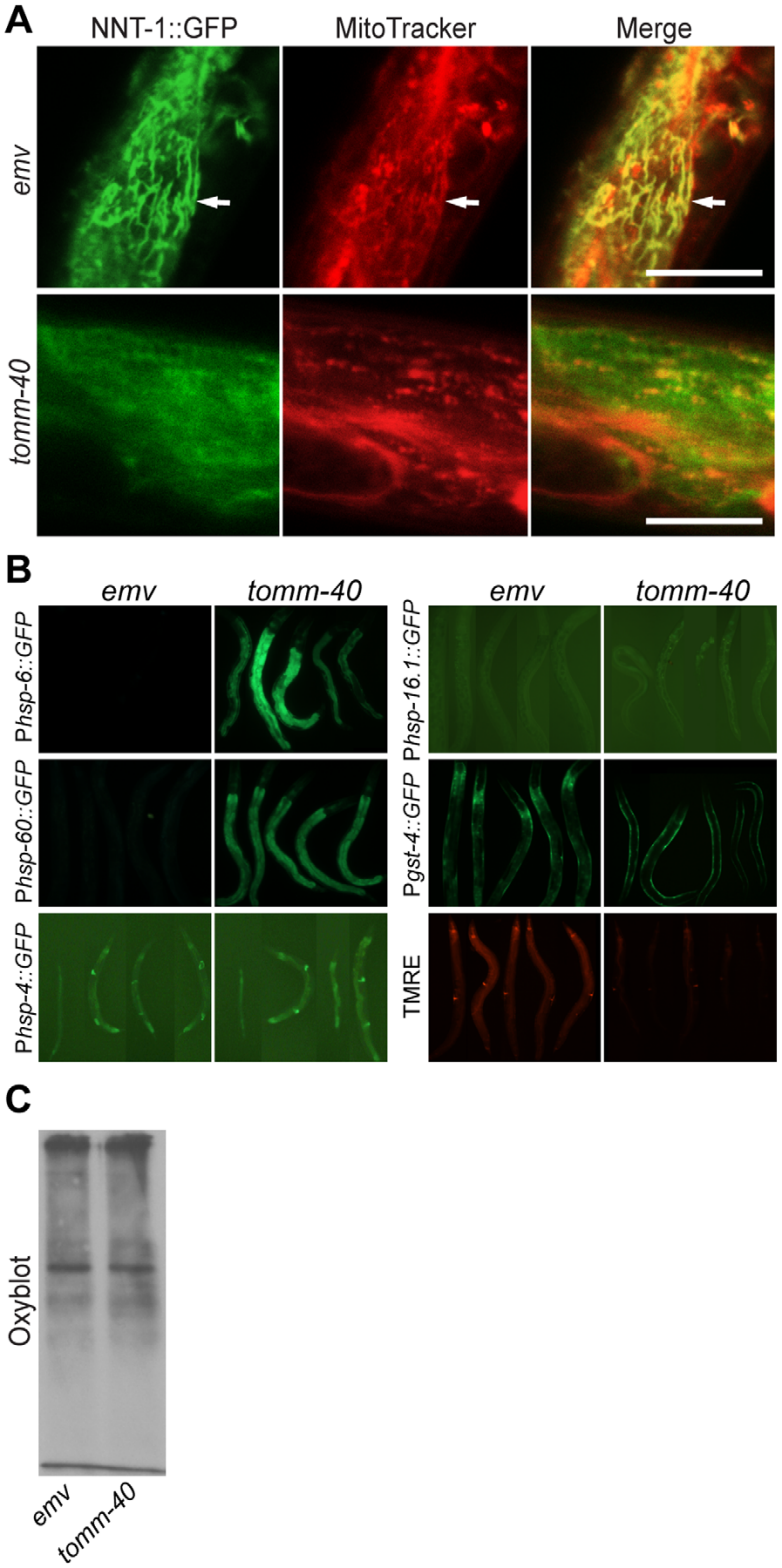
RNAi against TOMM-40 decreases mitochondrial function and protein import. (**A**) Confocal images of intestinal cells in RNAi treated animals expressing the truncated NNT-1::GFP protein and counterstained with MitoTracker red. Arrows indicate mitochondrial tubules labeled by both NNT-1::GFP and MitoTracker red. Scale bars are 20 µm. (**B**) Fluorescence optics imaging of RNAi-treated animals expressing P*_hsp-6_::gfp* (100 milliseconds); P*_hsp-60_::gfp* (1.01 seconds); P*_hsp-4_::gfp* (1.17 seconds); P*_hsp 16.2_::gfp* (100 milliseconds); P*_gst-4_::gfp* (300 milliseconds), or RNAi-treated animals stained with TMRE dye (300 milliseconds). (**C**) Image of a western blot showing the oxidized protein contents in whole worm protein extracts of *tomm-40(RNAi)* and *emv(RNAi)* larvae of similar ages, using the oxyblot assay. An equal amount of protein was loaded into each well. The gel shown is a representative of three individual experiments.

Studies in *S. cerevisiae* and *Neurospora crassa* show that TOM40 acts as a translocase subunit [21], [22]. To analyze *tomm-40-*dependent protein translocation *in vivo* in *C. elegans*, we made use of an *nnt-1::gfp* reporter construct [23]. NNT-1 (nicotineamide nucleotide transhydrogenase) is a nucleus-encoded protein that is predicted to be targeted to mitochondria (99.2% confidence) by the MitoProt program [24]. The *nnt-1::gfp* construct spans the promoter region of *nnt-1* and the first one and a half exons of its open reading frame, including the predicted mitochondrial targeting sequence. We found that NNT-1::GFP co-localized completely with red MitoTracker dye in intestinal and body wall muscle cells (Figure 4A, Figure S2). Treatment with *tomm-40(RNAi)* largely abolished the mitochondrial uptake of NNT-1::GFP in intestinal cells and shifted its localization to the cytosol. Thus a mitochondrially-targeted protein does not reach the mitochondrion when *tomm-40* is depleted. This is consistent with the observed role for its homolog in *S. cerevisiae* [15].

Perturbations affecting mitochondrial protein environment, like stochiometry changes or reduced mitochondrial chaperone function, induce a mitochondrial unfolded protein response. In *C. elegans* this response involves the chaperones mtHSP70/HSP-6 and HSP-60. The transcriptional reporters P*_hsp-6_::gfp* and P*_hsp-60_::gfp* are specifically upregulated in response to perturbations that affect mitochondrial protein handling [25]. Both reporters were distinctly upregulated by *tomm-40(RNAi)*, but not in “empty vector” *emv(RNAi)* controls (Figure 4B). Consistent with the RNAi results, the P*_hsp-6_::gfp* transgene was also greatly upregulated in *tomm-40(tm4574)* deletion mutants (Figure S3). This suggests that the mitochondrial protein milieu was disrupted when the level of functional TOMM-40 was reduced.

When mitochondria function normally they generate an electrochemical potential (ΔΨ) across the inner membrane of the organelle. ΔΨ is then used as the driving force for ATP synthesis from ADP+Pi. ΔΨ can be measured *in vivo* by the level of uptake of the dye tetramethylrhodamine ethyl ester (TMRE). Levels of TMRE uptake allow a distinction to be made between respiring and non-respiring mitochondria. There was markedly less TMRE staining in *tomm-40(peRNAi)* animals as compared to *emv(peRNAi)* controls, indicating a ΔΨ collapse across the inner mitochondrial membrane (Figure 4B).

Taken together, these results show that mitochondria are dysfunctional in animals with reduced levels of TOMM-40. Protein import was reduced, the protein milieu inside mitochondria was altered and ΔΨ had collapsed.

### *tomm-40(RNAi)* reduces insulin pathway activity

**Figure 5:**
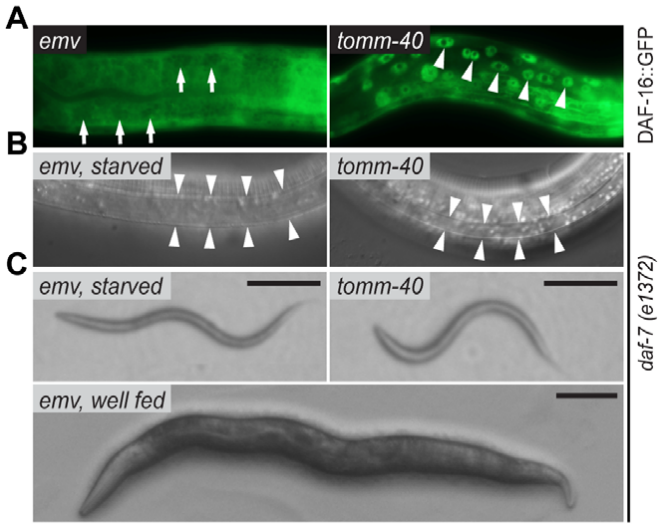
TOMM-40 is a positive regulator of insulin signaling. (**A**) Micrographs of RNAi treated worms, carrying an integrated *daf-16::gfp* transgene. Arrows indicate the absence of DAF-16::GFP in nuclei of intestinal cells in an empty vector RNAi [emv(RNAi)] fed animal. Arrowheads indicate nuclear DAF-16::GFP localization in a *tomm-40(RNAi)* animal. (**B**) Dauer-specific alae in a starvation-induced dauer larva and in a *tomm-40(RNAi)* semi-dauer larva. Arrowheads outline the alae. Both animals are homozygous for the *daf-7(e1372)* mutation. (**C**) A starvation-induced *daf-7(e1372)* dauer animal, a *tomm-40(RNAi)*-induced *daf-7(e1372)* semi-dauer animal and a well fed *daf-7(e1372)* adult. All experiments were performed at 20°C. Scale bars are 100 µm.

Signaling through the insulin receptor pathway is high when food is abundant and other general conditions favor reproductive growth [3]. High levels of signaling act to keep the downstream transcription factor DAF-16/FOXO in the cytoplasm of all cells. Conversely, low levels of signaling allow it to translocate to nuclei. This is detected *in vivo* with the use of functional *daf-16::gfp* transgenes [26], [27], [28]. In *tomm-40(RNAi)* animals raised on abundant food, DAF-16::GFP translocated to nuclei in 45% of the animals (n = 20), while it was always cytoplasmic in controls (Figure 5A), indicating lowered signaling through the insulin pathway.

In regulating entry into the dauer stage, the insulin pathway acts in parallel to the DAF-7/TGFβ pathway. The temperature sensitive *daf-7(e1372)* mutants rarely form dauers at 20°C but have a fully penetrant dauer-constitutive phenotype at 25°C. However, they form dauers at 20°C if insulin signaling is also weakly compromised at some level [29]. As a second test for the strength of insulin signaling, we therefore reduced TOMM-40 levels in *daf-7(e1372)* mutants. 42% (n = 124) of the *daf-7(e1372); tomm-40(RNAi)* animals entered a semi-dauer state at 20°C, while none of the *daf-7(e1372); emv(RNAi)* treated controls did so (n = 74) (Figure 5B). These semi-dauer animals had some of the dauer characteristics, while lacking others. In contrast to normal, starvation-induced dauers they were paler and they lacked oral plugs. On the other hand, these semi dauers displayed the dauer-specific nictitation behavior, had dauer alae (Figure 5B) and were SDS-resistant, as determined by the ability to survive exposure to 1% SDS for 20 minutes. 88% (n = 19) of the *daf-7(e1372); tomm-40(RNAi)* animals were SDS resistant, while none (n = 20) of the age-matched (L3) *daf-7(e1372); emv(RNAi)* controls were resistant. Semi-dauer animals were also seen in *daf-7(+); tomm-40(RNAi)* animals, but to a lesser extent.

Further, we performed *tomm-40(RNAi)* in *daf-2/insulin receptor* mutants. In contrast to the findings in the *daf-7(e1372)* background, *tomm-40(RNAi)* did not enhance dauer formation in *daf-2* mutants at 15°C. This suggests that mitochondrial dysfunction affects processes in the *daf-2/insulin receptor* pathway and in parallel to the TGFβ pathway. This also suggests that mitochondrial dysfunction can lead to a sensitized insulin signaling state, in which further perturbations can be easily assessed.

Taken together these three tests show that insulin signaling is weaker when TOMM-40 levels are reduced. Since high levels of insulin signaling are needed for reproductive (non-hibernating) growth, the larval arrest seen in *tomm-40(RNAi)* animals might be due in part to a defect in insulin signaling.

### Investigation of other stress responses in *tomm-40* depleted animals

In addition to changes in insulin signaling strength, the nuclear localization of DAF-16::GFP can be due to elevated levels of different kinds of stress such as oxidative stress, heat shock and ER stress. To investigate whether these factors may have contributed to the nuclear localization of DAF-16 in *tomm-40(RNAi)* animals, we used specific gene expression reporters and biochemical assays to address this issue.

Perturbations of mitochondrial functions can give rise to increased levels of oxidative damage to cells and trigger oxidative stress responses [30], [31], [32]. To test if oxidative stress levels were elevated by *tomm-40(RNAi)*, we first made use of the the P*_gst-4_::gfp* transgene which is known to be upregulated by increased levels of intracellular oxidative stress [33]. Using this transgene, we were unable to detect any increase of the GFP signal upon *tomm-40(RNAi)* compared to *emv(RNAi)*. Instead we noted a slight decrease in P*_gst-4_::gfp* expression in the intestine and in the nerve ring in *tomm-40(RNAi)* animals (Figure 4B).

As a test for oxidative damage, we measured total levels of oxidized proteins using a commercially available oxyblot kit. We measured the relative amount of carbonylated proteins which result from protein oxidation in *emv(RNAi)-*treated and *tomm-40(RNAi)*-treated larvae of similar ages (Figure 4C). The levels of carbonylated proteins in worms is an indirect measure of its redox status, since proteins that are oxidized become irreversibly carbonylated [34] and since the carbonylation status in worms is not reset until embryogenesis [35]. Using this assay we could detect no significant difference between lysates from *emv(RNAi)* and *tomm-40(RNAi)* animal. Taken together, the P*_gst-4_::gfp* transgene and the oxyblot results indicate that *tomm-40(RNAi)* does not induce oxidative stress or oxidative damage significantly.

We next assayed for endoplasmic reticulum stress and cytosolic stress using specific reporters. Neither the endoplasmic reticulum (ER) stress reporter P*_hsp-4/BiP_::gfp*, nor the cytosolic stress reporter P*_hsp-16.2_::gfp* was upregulated in *tomm-40(RNAi)* animals (Figure 4B). Thus we conclude that oxidative stress, ER stress and general cytosolic stress are not greatly induced in *tomm-40(RNAi)* animals and that these forms of stress conditions are not likely to play a significant role in promoting nuclear localization of DAF-16::GFP.

### *tomm-40(peRNAi)* reduces DAF-28 secretion

The insulin/IGF type ligand DAF-28 is required to promote non-dauer development and in its absence worms enter the dauer stage inappropriately. In *svIs69* worms, harboring an integrated *daf-28::gfp* array [8], DAF-28::GFP is expressed in sensory neurons and is secreted into the pseudocoelom. There it is taken up by the coelomocytes, which are cells specialized to take up material from the pseudocoelomic fluid. Since DAF-28 is not expressed in coelomocytes, any DAF-28::GFP accumulating in these cells is due to uptake of the secreted protein [8]. Under growth-promoting conditions, adults accumulate enough DAF-28::GFP in their coelomocytes to give a distinct GFP-pattern, which is seen in 100% of wild type animals [8]. The kinetics of secretion are such that very little DAF-28::GFP is detectable in coelomocytes in *svIs69* bearing larvae. As a result, the secretion assay was carried out in adults using the weaker peRNAi treatment to bypass the larval arrest.

**Figure 6:**
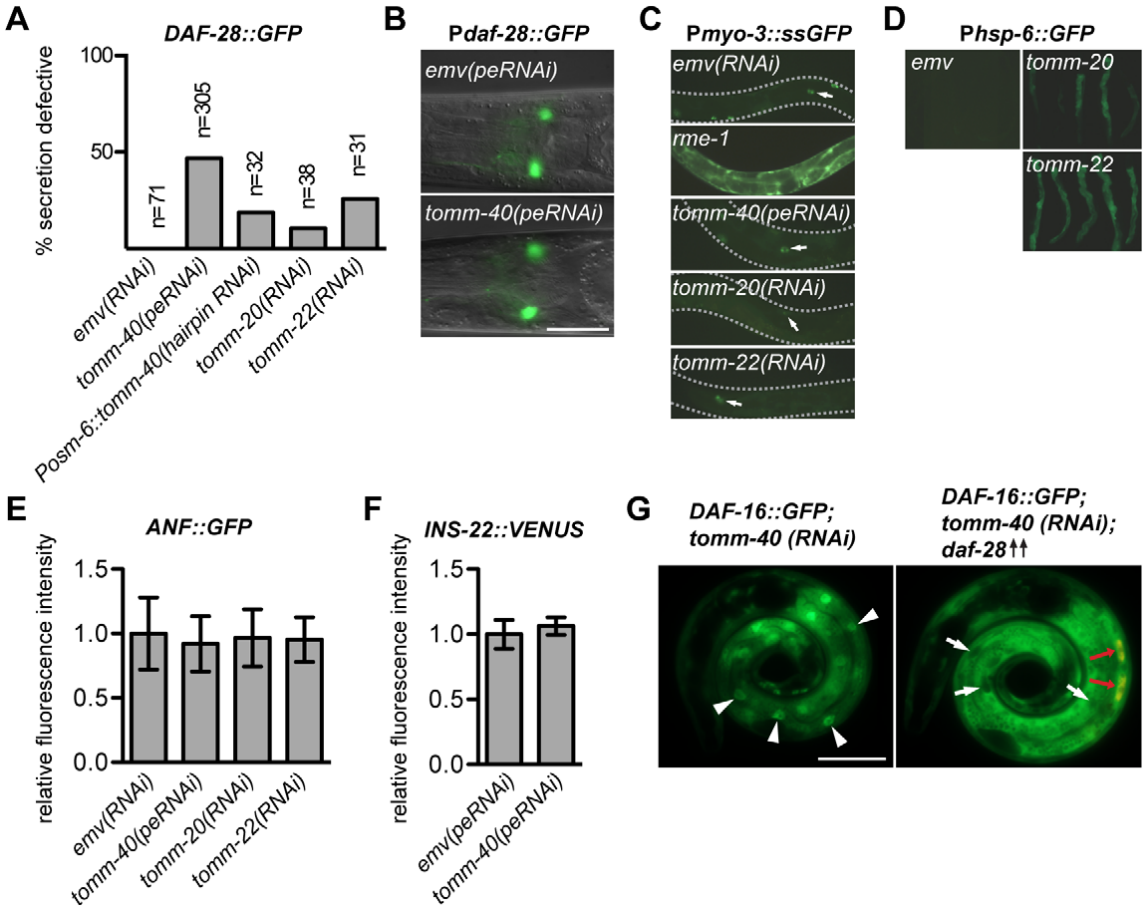
TOMM-40 promotes insulin secretion. (**A**) Adults, carrying an integrated *daf-28::gfp* transgene (*svIs69)*, were analyzed for coelomocyte GFP content. Absent or severely reduced GFP content (a maximum of two faintly fluorescing coelomocytes) was scored as secretion defective. (**B**) Neuronal expression of GFP from the transcriptional reporter transgene P*_daf-28_::gfp* in *tomm-40(peRNAi)* and *emv(peRNAi)* treated animals, imaged with fluorescence optics. (**C**) Fluorescence optics of peRNAi treated animals of the *arIs37* strain, carrying a P*_myo-3_::ssgfp* transgene that expresses ssGFP in body wall muscle, from where it is secreted into the pseudocoelom. Coelomocyte sequestration of GFP indicates functional coelomocyte endocytosis. *rme-1(b1045)* is a previously characterized endocytosis defective mutant [39]. Arrows indicate GFP labeled coelomocytes. (**D**) Fluorescence micrographs of *emv, tomm-20* and *tomm-22(RNAi)*-treated animals carrying the P*_hsp-6_::gfp* reporter. (**E–F**) Relative pixel intensity plots of coelomocyte GFP contents in peRNAi or RNAi treated adults carrying (**E**) an *Anf::gfp* transgene or (**F**) an *ins-22::venus* transgene. The strongest fluorescing coelomocyte in the posterior most pair was scored. The mean value of the pixel intensity in *emv* was set to 1 in each experiment. Error bars represent +/− mean standard deviations from three independent experiments. (**G**) Micrographs of RNAi treated sibling animals, carrying either the integrated *daf-16::gfp* transgene only, or both the integrated *daf-16::gfp* transgene and an extra chromosomal P*_daf-28_::daf-28* transgene for DAF-28 overexpression. Presence of the P*_daf-28_::daf-28* transgene is indicated by a coelomocyte RFP co-injection marker (red arrows). White arrowheads indicate nuclear DAF-16::GFP localization in a *tomm-40(RNAi)* animal. White arrows indicate the absence of DAF-16::GFP in nuclei of intestinal cells in a *tomm-40(RNAi); daf-28(++)* animal. Scale bars are 25 µm.

Since signaling through the insulin receptor was low in *tomm-40(RNAi)* animals, we investigated whether this might be caused by defective DAF-28 secretion. In *daf-28::gfp; tomm-40(peRNAi)* animals there was a substantial decrease in the number of animals with GFP-labeled coelomocytes (Figure 6A). The defect was not due to decreased transcription of *daf-28*, since we could not detect any down regulation of the transcriptional reporter P*_daf-28_::gfp* in *tomm-40(peRNAi)* animals (n = 18) (Figure 6B). Further, we never observed any reduced expression in neurons from the functional *svIs69* transgene upon *tomm-40(peRNAi)*. We next wished to investigate whether the DAF-28 secretion defect was due to secondary effects of the mitochondrial inactivation, or due to a neuron specific effect, since neurons are refractory to feeding RNAi against some genes [36]. Expression of hairpin RNAi from transgenes is in such cases an effective strategy for silencing neuronal gene message [36]. To silence *tomm-40* expression in neurons, we therefore expressed *tomm-40* hairpin RNAi under the promoter of *osm-6*, which is specifically expressed in ciliated neurons [37]. Like *tomm-40(peRNAi)*, P*_osm-6_::tomm-40(hairpin RNAi)* rendered animals unable to secrete DAF-28::GFP normally (Figure 6a).

Given the broad range of TOMM-40 expression, it was possible that coelomocyte function could be affected in *tomm-40(peRNAi)* animals. In that case, the effect seen with DAF-28::GFP would reflect a defect in the uptake of secreted proteins by coelomocytes rather than a secretion defect. To test this we investigated whether coelomocytes in *tomm-40(peRNAi)* animals were competent to take up and sequester a secreted form of GFP (ssGFP). A P*_myo-3_::ssgfp*-bearing transgene (*arIs37)* is expressed in body wall muscles, from where the ssGFP is secreted into the pseudocoelom. It is then taken up and sequestered by coelomocytes, leaving very little ssGFP in the pseudocoelum [38]. Mutants with defective endocytosis in coelomocytes accumulate large amounts of ssGFP in the pseudocoelum [Figure 6B and [39]]. Using these transgenic worms we found no differences in uptake of ss::GFP by coelomocytes between *tomm-40(peRNAi)* (n = 31) and *emv(peRNAi)* (n = 30) animals (Figure 6B). We conclude that the reduced levels of DAF-28::GFP in coelomocytes in *tomm-40(peRNAi)* was not due to a coelomocyte endocytosis defect.

### DAF-28 overexpression rescues the insulin signaling defect in *tomm-40* depleted animals

To investigate whether the diminished insulin signaling downstream of the DAF-2 receptor was due to defective DAF-28 secretion or to other secondary effects, we over expressed P_daf-28_::*daf-28* on an extrachromosomal array in animals expressing *daf-16::gfp.* Since extrachromosomal arrays are unstable during meiosis, the progeny of a transgenic mother will have transgenic (*daf-28* overexpressing) and non-transgenic (wild-type *daf-28* levels) siblings on the same culture plate. By analyzing siblings that were subjected to *tomm-40(RNAi)* on the same plate, we observed that 70.6% of the animals that did not overexpress *daf-28* (n = 17) had nuclear localization of DAF-16::GFP. Of the animals that overexpressed *daf-28*, only 14.8% had nuclear DAF-16::GFP (n = 27). Thus increased levels of DAF-28 can bypass the insulin signaling defect in animals with defective mitochondria.

### Defective DAF-28 secretion is a general feature of worms depleted of TOMM complex function

Based on the studies of TOMM complex functions in other organisms [22], [40], *C. elegans* TOMM-40 is likely to be part of a complex, where it acts along with other components, such as Tom20 and Tom22, to import proteins into the mitochondria. We performed RNAi against their worm homologues, *tomm-20* (cosmid name: F23H12.2) and *tomm-22* (cosmid name: W10D9.5), to test whether the phenotypes elicited by their inactivation would resemble that of *tomm-40(RNAi)* animals. We found that RNAi against these genes did not produce the highly penetrant larval arrest phenotype seen in *tomm-40(RNAi)* worms. However, RNAi against either *tomm-20* or *tomm-22* induced the mitochondrial unfolded protein response, indicating a perturbed mitochondrial protein handling (Figure 6C). Further, inactivation of *tomm-20* or *tomm-22* caused a defect in DAF-28 secretion (Figure 6A), although to a less extent than in *tomm-40(peRNAi)*. As with *tomm-40(peRNAi)*, coelomocytes in *tomm-20(RNAi)* (n = 19) and *tomm-22(RNAi)* (n = 24) animals were competent for endocytosis (Figure 6B). In summary these results demonstrate that the process under study is not a unique property of TOMM-40, but rather the result of defect in the TOMM complex.

### Mitochondrial dysfunction does not affect the secretion of ANF and INS-22 neuropeptides

We next asked whether the secretion defect in TOMM-deficient animals was specific to DAF-28 or if it could also affect secretion of another insulin-type neuropeptide or a non-insulin neuropeptide. An established model of non-insulin neuropeptide secretion makes use of a heterologous fusion protein, ANF:GFP, under control of the pan-neural *aex-3* promoter. The fusion protein localizes to dense core vesicles in neuronal cells, from where it is secreted into the pseudocoelom [41]. Like DAF-28::GFP, ANF::GFP is also taken up by coelomocytes. Since we detected no obvious differences in coelomocyte GFP contents, we instead used pixel intensity measurements for increased sensitivity. Secretion and uptake of ANF::GFP was unaffected in *tomm-40(peRNAi)* animals as well as in *tomm-20(RNAi)* and *tomm-22(RNAi)* (Figure 6E). Similar to ANF, the insulin-type neuropeptide INS-22 is secreted from dense core vesicles into the body cavity, where it is taken up by coelomocytes [42]. Using pixel intensity measurements in coelomocytes we were unable to detect a difference between *emv(peRNAi)* and *tomm-40(peRNAi)* animals in secretion and coelomocyte uptake of INS-22::VENUS (Figure 6F, Figure S4).

Thus we were able to discriminate between the secretion patterns of different dense core vesicle requiring neuropeptides based on their sensitivity to perturbations of the TOMM complex. The effect of mitochondrial dysfunction on DAF-28::GFP secretion is therefore not due to a general neuropeptide secretion defect or to a defect in the dense core vesicle secretion machinery.

## Discussion

This work demonstrates that TOMM-40 is essential for mitochondrial function in *C. elegans*. Its absence severely reduces mitochondrial function, and causes a larval growth arrest. Using sensitized genetic backgrounds and specific reporters we find that insulin signaling is compromised upon depletion of *tomm-40* and that this is due to a decrease in DAF-28 secretion. The secretion defect is not general in nature because these animals are able to secrete ANF::GFP and INS-22::VENUS, two other dense core vesicle residing neuropeptides, normally.

The mitochondrial defects inflicted upon inactivation of *tomm-40* are likely caused by a decreased protein import capacity of mitochondria, which we observed with the NNT-1::GFP fusion. A defective import of cytosolic proteins into mitochondria presumably causes stochiometric changes. Changes in the stochiometry of mitochondrial proteins are known to evoke expression of the mitochondrial unfolded protein response, as are elevations in mitochondrial ROS levels and misfolding of mitochondrial proteins [25].

Worms very rarely became adults in the stronger form of RNAi against *tomm-40*. Instead they arrested as larvae, mostly between the 1^st^ and 3^rd^ larval stage, which is consistent with the elevated energy demand at the L3/L4 transition [43]. The increase in biosynthetic activity at this transition invokes a substantial energy demand, which is reflected by a sharp increase in the number of mitochondria at this stage [44]. It is possible that cytosolic glycolysis can supply sufficient energy for completion of embryogenesis and for progression through the first three larval stages in *tomm-40*-depleted animals, but fails to do so in the energy-demanding L3/L4 transition. The L1 arrest phenotype is also seen in other situations when insulin signaling is compromised. Worms that hatch in the absence of food arrest as L1s, as do *daf-2/insulin receptor* mutants [45] and *asna-1* mutants [8].

In contrast to the developmental defects in *tomm-40*, we did not observe any growth defects or sterility when *tomm-20* or *tomm-22* were inactivated. This was not due to RNAi-insensitivity since the mitochondrial stress response was strongly evoked. *tomm-20* and *tomm-22*-depleted animals also showed insulin secretion defects, but to a lesser extent than in *tomm-40(peRNAi)*. The only phenotype that was at equal levels in all three cases was the induction of mitochondrial stress. Since we did not find a correlation between the extent of mitochondrial stress and the extent of the DAF-28 secretion defect, the stress response appears to be uncoupled from DAF-28 secretion.

The differences in phenotypes between *tomm-40* and the other two *tomm* genes is not unexpected and is in keeping with work in *S. cerevisiae* and mammalian cells showing that TOMM-40 is the central element of the translocase [14] and that other TOMM proteins like TOMM-20 and TOMM-22 play subsidiary roles, in recognizing preproteins destined for import and in stabilizing the complex [16]. It therefore appears that *tomm-40* is the one gene which when inactivated will cause a loss-of-organelle function by preventing import of nucleus-encoded structural proteins and proteins needed for transcription and translation of the mitochondrial genome. This is consistent with the finding that inactivation of only the mitochondrial genome by ethidium bromide treatment causes the milder phenotypes of slow growth and reduced brood size [25]. Inactivation of *tomm-40* therefore enabled us to evaluate the contribution of mitochondria to insulin secretion and signaling unambiguously.

Two findings suggest to us that insulin signaling is weaker in *tomm-40* depleted animals. First, DAF-16::GFP was in nuclei in *tomm-40(RNAi)* animals (Figure 5A). Second, *tomm-40(RNAi)* enhanced the dauer defect of *daf-7/TGFβ* mutants, but not of *daf-2/insulin receptor* mutants (Figure 5B,C). It is very likely that the primary defect that caused a lowered insulin signaling level in *tomm-40(RNAi)* animals was a decrease in DAF-28 secretion because overexpression of DAF-28 in *daf-16::gfp; tomm-40(RNAi)* animals rescued the nuclear localization of *daf-16::gfp* (Figure 6G). We speculate that there can be a basal level of DAF-28 secretion that is mitochondria-independent and that this level may be greatly increased upon overexpression of DAF-28. One other reason for the rescue could be an increased loading of DAF-28 into dense core vesicles, so that any one vesicle that is secreted contains more DAF-28. The effect of *tomm-40* depletion on DAF-28 secretion is likely to be in the neurons because expression of a *tomm-40* hairpin RNAi construct in ciliated head neurons (a subset of which express *daf-28*) also reduces DAF-28::GFP secretion (Figure 6A).

While DAF-16::GFP can be driven into nuclei because of various stresses such as oxidative stress, pathogen load and heat shock, we do not think that these are the reasons for the nuclear localization of DAF-16::GFP in *tomm-40(RNAi)* animals. Using specific stress reporters, we could not detect an increase in oxidative stress, ER stress or general cytosolic stress (Figure 4B). A direct estimation of the levels of oxidized proteins in *tomm-40(RNAi)* animals also indicated that there was no increase in oxidative stress (Figure 4C).

DAF-28 secretion was compromised and the insulin pathway activity downstream of daf-2/insulin receptor was lowered in *tomm-40(peRNAi)* animals. Notably, the DAF-28::GFP fluorescence in coelomocytes is rarely detected before the adult stage, presumably because the rate of secretion is sufficient only in adults. As a result, a weaker form of RNAi against *tomm-40* had to be performed to bypass the larval arrest. Therefore the DAF-28::GFP secretion defect observed in *tomm-40(peRNAi)* is most likely an underestimate of the true magnitude of the defect since much *tomm-40* activity is likely to persist. By contrast this type of treatment had no discernable effect on the secretion of two other dense core vesicle-associated neuropeptides, ANF::GFP and INS-22::VENUS.

Of the many insulins expressed in *C. elegans*, DAF-28 has some features of an insulin neuropeptide that plays a role in regulating metabolic homeostasis. DAF-28 acts as an agonist to the *DAF-2/insulin receptor*, thereby promoting reproductive growth and preventing dauer stage entry in nutritionally replete conditions [7], [46]. While its regulation is complex and incompletely understood, it is notable that, like human insulin, its expression is high when food levels are high and low when food levels are low. *daf-28* is regulated transcriptionally by the dauer pheromone, during ageing [7] and by *daf-11/guanylyl cyclase* [7], [47]. However, it has been noted that the magnitude of transcriptional control does not appear sufficient to modulate dauer formation. This work demonstrates that DAF-28 secretion is sensitive to mitochondrial function and that failure to secrete DAF-28 is associated with inappropriate entry into the dauer diapause. In that way, our findings indicate that mitochondrial activity seems to represent an additional level of control over DAF-28 function.

RNAi treatment often does not affect neuronally expressed genes in *C. elegans*. However, this does not seem to be the case for *tomm-40*, because *tomm-40(peRNAi)* affected the secretion of neuronally expressed DAF-28. It is therefore very likely that *tomm-40(peRNAi)* also affects neurons that express ANF::GFP (Figure 6E) and INS-22::VENUS (Figure 6F). We conclude that a severe effect of *tomm-40(peRNAi)* on DAF-28::GFP reflects a greater dependence of DAF-28 on mitochondrial activity, which is consistent with the case for human insulin. The secretion of DAF-28 also mimics that of mammalian insulins in that it is dependent on the ATPase *asna-1* [8] and the dense core vesicle component *unc-31/CAPS* [48]. We have demonstrated one more level of similarity. Just as in the case of human insulin, DAF-28 secretion is sensitive to mitochondrial function. Hence, several factors controlling DAF-28 secretion in *C. elegans* are similar to those controlling insulin secretion in mammals. More knowledge of the mechanisms underlying DAF-28 secretion is therefore of interest to the field of insulin secretion research in a larger context.

## Materials and Methods

### Plasmids

pVB463OB: The full length *tomm-40* cDNA from ATG to the stop codon was inserted as a BamHI/XhoI fragment into the feeding RNAi vector L4440. This was used for feeding RNAi.

pVB488OB: A region spanning from 1.8 kb of the promoter region and 16 bp of the first exon of *tomm-40* was inserted as a HindIII/PstI fragment into the GFP vector pPD95.77 plasmid to make a transcriptional GFP reporter.

pVB518OB: Using an oligo dT primed first strand cDNA reaction from total *C.elegans* RNA, the full length *tomm-40* cDNA was amplified using primers with flanking AgeI sites. The amplified cDNA, which was designed to lack the stop codon, was inserted between the *tomm-40* promoter and the gfp coding region in pVB488OB at the AgeI site to yield pVB518OB which contains a full length *tomm-40::gfp* fusion construct. The cDNA was sequenced and found to contain no PCR-generated errors and to be in frame with *gfp*.

pVB299GK: A 2.5 kb fragment containing the promoter region of *osm-6* was inserted as a SphI/XbaI fragment into the pPD49.26 plasmid.

pVB559MA: *tomm-40* genomic DNA was amplified using primers with flanking NheI sites corresponding to the start and to the stop of the gene, but lacking the start and stop codons. This fragment was inserted as an NheI/NheI fragment into the pVB299GK plasmid to generate P*_osm-6_::tomm-40*, where *tomm-40* was inserted in a 5′to 3′orientation. The orientation was confirmed by sequencing.

pVB560MA: We used the same method as with pVB559MA, but instead selelected a clone where *tomm-40* was inserted in a 3′to 5′orientation. The orientation was confirmed by sequencing.

### Strains

The following *C. elegans* mutants and transgenes used in this study are described in WormBase (www.wormbase.org, release WS211): Wild type Bristol strain (*N2*), *daf-16::gfp(zIs356)*, *daf-7(e1372)*, *daf-2(e1370), anf::gfp(oxIs180)*, P*_hsp-4_::gfp(zcIs4)*, P*_hsp-6_::gfp(zcIs13)*, P*_hsp-60_::gfp(zcIs9)*, P*_hsp-16.2_::gfp(dvIs70)*, P*_gst-4_::gfp(zcIs19)* and *P_unc-129_::ins-22::venus(nuIs196)*. Other strains were: *daf-28::gfp(svIs69)* [8], P*_daf-28_::gfp(svEx436)* [8], P*_myo-3_::ssgfp(arIs37)* [38] *nnt-1::gfp(svEx127)* [23]. *arIs37; rme-1(b1045)* was generated by standard crossing techniques. The P*_tomm-40_::gfp* and P*_tomm-40_::tomm-40::gfp*-bearing strains were generated by injection of pVB488OB and pVB518OB at 50 ng/µL respectively. For both plasmids, transgenic lines were generated by co-injecting of each plasmid with the *rol-6(d)* plasmid at a concentration of 50 ng/µL. The *daf-16::gfp; daf-28(++)* strain was generated by injecting the pVB288GK plasmid [8] into the *daf-16::gfp(zIs356)* strain. The pVB288GK plasmid, which contains wild type daf-28 under 3.4 kb of its own promoter, was co-injected with a P*_unc-122_::RFP* (coelomocyte::RFP) marker [49] (Addgene plasmid 8938). Both plasmids were injected at a concentration of 50 ng/µl. The *daf-28::gfp(svIs69)*; P*_osm-6_::tomm-40(hairpin RNAi)* strain was generated by co-injecting pVB559MA and pVB560MA at a concentration of 1 ng/µL each together with the co-injection marker plasmid *rol-6(d)* at a concentration of 50 ng/µL.

### Analysis of tomm-40(tm4574)

The *tomm-40(tm4574)* mutant was outcrossed five times and balanced with the *mnC1(dpy-10 unc-52)* balancer chromosome [50]. The primers ATACACCACCAACAGTCCTG and GTGCTGCGAATAAACCCTTC that were recommended by the NBP, Tokyo were used in single worm PCR to genotype the mutants. These primers allowed us to distinguish among wild-type, heterozygotes and homozygous mutants. *tomm-40(tm4574)* mutants segregating from *tomm-40/mnC1* mothers were identified on plates by allowing 6 mothers to lay eggs for 2–3 hours and then killing all the L4 larvae that emerged after incubation for 40 hours. Motile L2 larvae were picked to a second plate and incubated for a further 24 hours to confirm that these were growth arrested. They were then used for microscopy or single worm PCR. P*_hsp-6_::gfp(zcIs13); tomm-40(tm4574)/mnC1* animals were handled in the same way to identify *tomm-40* mutants bearing the P*_hsp-6_::gfp* transgene.

### RNAi

*Embryonic RNAi*: The bacterial strain HT115 was transformed to carbenicillin resistance with pVB463OB for RNAi against *tomm-40*. The *tomm-20* and *tomm-22* RNAi clones were from the Ahringer lab library of clones [51]. HT115 bacteria bearing the L4440 plasmid served as the empty vector (emv) control [52]. Feeding RNAi was done as described elsewhere [53]. In each experiment, the progeny from three hermaphrodites were scored for phenotypes elicited by RNAi. *Postembryonic RNAi*: Gravid hermaphrodites were treated with sodium hypochlorite solution to release embryos. After washing three times in M9, 50–200 embryos were put on each RNAi plate, seeded with either pVB463OB or emv expressing bacteria and examined for phenotypes after hatching and larval growth.

### Feeding behavior

The number of pharyngeal pumps/minute in arrested animals was counted using a Leica MZFLIII dissecting microscope at 40× magnification. Wild type worms, which were arrested as L1s through hatching in the absence of food served as the positive control. They were fed with *emv*-containing bacteria 30 minutes before analysis to start feeding and were then compared with worms fed with pVB463OB containing bacteria.

The ability to ingest food was measured using fluoresbrites 0.2 mM fluorescent microspheres (Polyscience, Inc., Warrington, PA, USA). Beads were mixed with RNAi bacteria suspensions at 1:50 dilution and seeded onto RNAi plates, followed by IPTG induction over night. Worms were first treated with feeding RNAi as before, and then incubated for 30 minutes on the lawn of bacteria mixed with fluorescent beads. Ingestion of these bacteria-sized beads was visualized at 63× magnification under a Leica DMRB fluorescence microscope. Control animals were arrested as before and then allowed to start feeding on a lawn of *emv* bacteria 30 minutes before incubation on the corresponding bacterial lawn with fluorescent beads.

### Insulin signaling and neuropeptide secretion

DAF-16::GFP: Worms carrying an integrated *daf-16::gfp* transgene array [28] were treated with feeding RNAi as above. Micrographs were taken at 63× or 100× magnification using a Leica DMRB fluorescence microscope. *tomm-40* and *emv(RNAi)* treated *daf-16::gfp* animals were kept on microscope slides for no more than 10 minutes to avoid artifacts due to stressful conditions.

DAF-28::GFP uptake by coelomocytes was scored in adult worms, carrying the integrated *daf-28::gfp* array *svIs69* [8]. The animals were scored as defective in DAF-28::GFP secretion if they had no GFP-labeled coelomocytes or if the coelomocytes had very faint GFP fluorescence. They were scored as secretion competent if they possessed one or more brightly fluorescing coelomocyte [8].

The ANF::GFP content in coelomocytes was measured in peRNAi treated animals as described previously [41]. Briefly, fluorescence images of the posterior most coelomocytes (either ccDL or ccDR) were captured using a fixed exposure time of 360 milliseconds. Images were converted to grayscale using Photoshop software and the pixel intensity was then measured with the aid of the NIH ImageJ software.

### Mitochondrial imaging and physiology

To assess the strength of the electrochemical gradient of mitochondria, *tomm-40(peRNAi)* treated animals were grown at 20°C on plates containing 30 µM tetramethylrhodamine ethyl ester (TMRE) (Invitrogen, Carlsbad, CA, USA) for 16 hours as described previously [40]. Animals were washed in M9 buffer and anesthetized in 0.5 mM levamisole. Micrographs were captured at 20× magnification using a Leica DMRB fluorescence microscope.

MitoTracker counterstainings of mitochondria were done as described elsewhere [20], by growing worms on plates containing 1.25 mM MitoTracker red CMXRos (Invitrogen, Carlsbad, CA, USA). Confocal images were captured using a Nikon Eclipse C1 microscope with excitation/emission wavelengths at 488/515 nm for GFP and at 543/605 nm for MitoTracker. Fluorescence images were captured using a Leica DMRB fluorescence microscope.

### Assay for oxidized proteins

Mothers were treated from the L4 stage and onwards with feeding RNAi against *tomm-40* or the L4440 plasmid (emv) as a control. The mothers were allowed to lay eggs for 24 hours, and were then removed. The larval offspring was washed carefully in M9 buffer to remove bacteria, and then lysed with a bullet blender (Next Advance, Averill Park, NY, USA) in Nonidet P40 buffer (150 mM sodium chloride, 1.0% NP-40, 50 mM Tris pH 8.0, 1× protease inhibitor cocktail). Worm debris was removed by centrifugation and protein levels in the remaining worm lysates were measured using the Bio-Rad DC protein assay (Bio-Rad, Hercules, CA, USA). Samples from each experiment were then normalized to protein levels and treated according to the manufacturers instructions for the Oxyblot kit (Millipore, Billerica, MA, USA).

## Supporting Information

Figure S1 TOMM-40::GFP localizes to mitochondria. Fluorescence imaging of a body wall muscle cell in an animal transgenic for the *tomm-40::gfp* (pVB518OB) plasmid. (A) TOMM-40::GFP localized in stripy patterns corresponding to red MitoTracker dye in (B) and (C). The dashed squares indicate the areas enlarged in (A'-C'), where arrows indicate TOMM-40::GFP localized around a red MitoTracker foci, corresponding to the matrix of a mitochondion.
(1.71 MB TIF)

Figure S2 TOMM-40 is required for NNT-1::GFP to accumulate in mitochondria. Whole body confocal images of RNAi treated animals, transgenic for a truncated *nnt-1::gfp* construct and counterstained with MitoTracker. Dashed squares represent the portions that are enlarged in Figure 4A. Scale bars are 20 μm.
(2.84 MB TIF)

Figure S3 The mitochondrial stress response is evoked in *tomm-40(tm4574)* mutants. Fluorescence optics imaging of P*_hsp-6_::gfp* and P*_hsp-6_::gfp; tomm-40(tm4574)* animals. Images were captured at 1.01 seconds exposure time for P*_hsp-6_::gfp* animals and at 0.1 seconds for P*_hsp-6_::gfp; tomm-40(tm4574)* animals.
(0.20 MB TIF)

Figure S4 Accumulation of DAF-28::GFP and INS-22::Venus in coelomocytes. (A) Animals of the *daf-28::gfp(svIs69)* strain, treated with *tomm-40(peRNAi)* or *emv(RNAi)*. Top panels are DIC representations of the fluorescence images below. (Left) An *emv(peRNAi)* animal, with vibrantly GFP-labeled coelomocytes. This animal was scored as secretion competent. (Right) A *tomm-40(peRNAi)* animal, with absence of any GFP-labeled coelomocyte. This animal was scored as secretion defective. Arrows indicate coelomocytes. The scale bar is 25 μm. (B) Picture representation of the coelomocytes that were measured for pixel intensity in Figure 6F. (Top) A panel showing 33 coelomocytes, from individual *emv(peRNAi)* worms, containing INS-22::VENUS and (below) 34 coelomocytes, from individual *tomm-40(peRNAi)* worms, containing INS-22::VENUS.
(0.67 MB TIF)
