# Supplementary figures and images for: Mitochondrial Function Is Required for Secretion of DAF-28/Insulin in C. elegans
Source: PLoS One. 2011 Jan 17;6(1):e14507. doi: 10.1371/journal.pone.0014507 (PMC3022011; doi:10.1371/journal.pone.0014507)

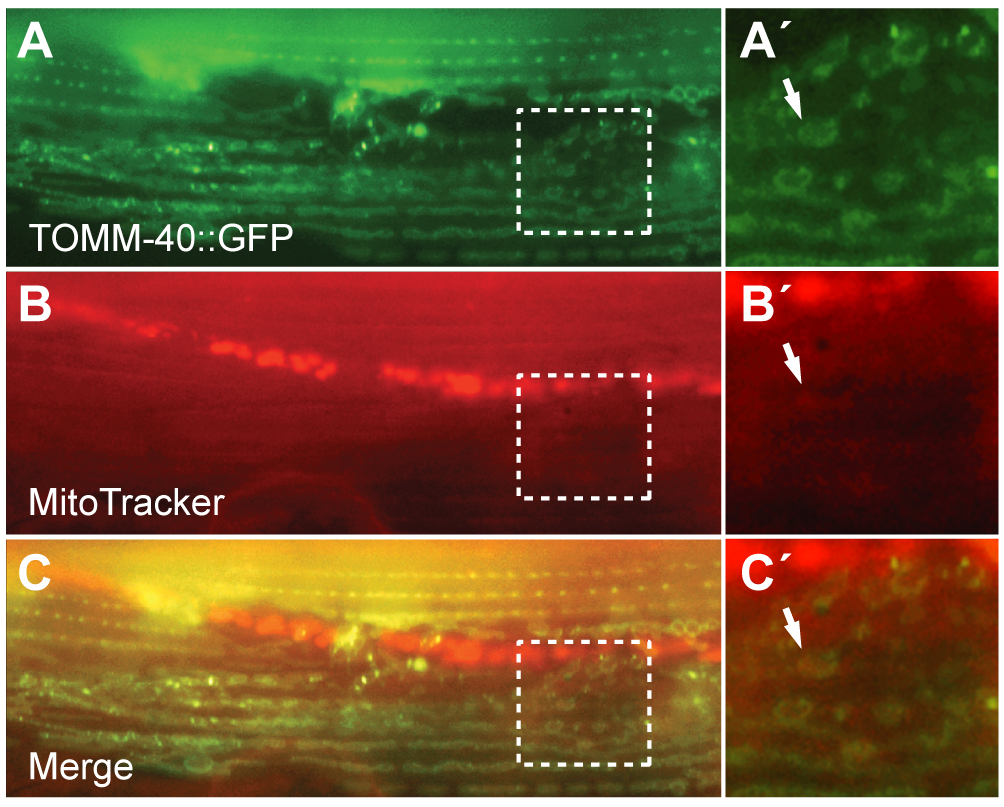

Supplement: Figure S1 — TOMM-40::GFP localizes to mitochondria. Fluorescence imaging of a body wall muscle cell in an animal transgenic for the tomm-40::gfp (pVB518OB) plasmid. (A) TOMM-40::GFP localized in stripy patterns corresponding to red MitoTracker dye in (B) and (C). The dashed squares indicate the areas enlarged in (A'-C'), where arrows indicate TOMM-40::GFP localized around a red MitoTracker foci, corresponding to the matrix of a mitochondion. (1.71 MB TIF) [file pone.0014507.s001.tif]

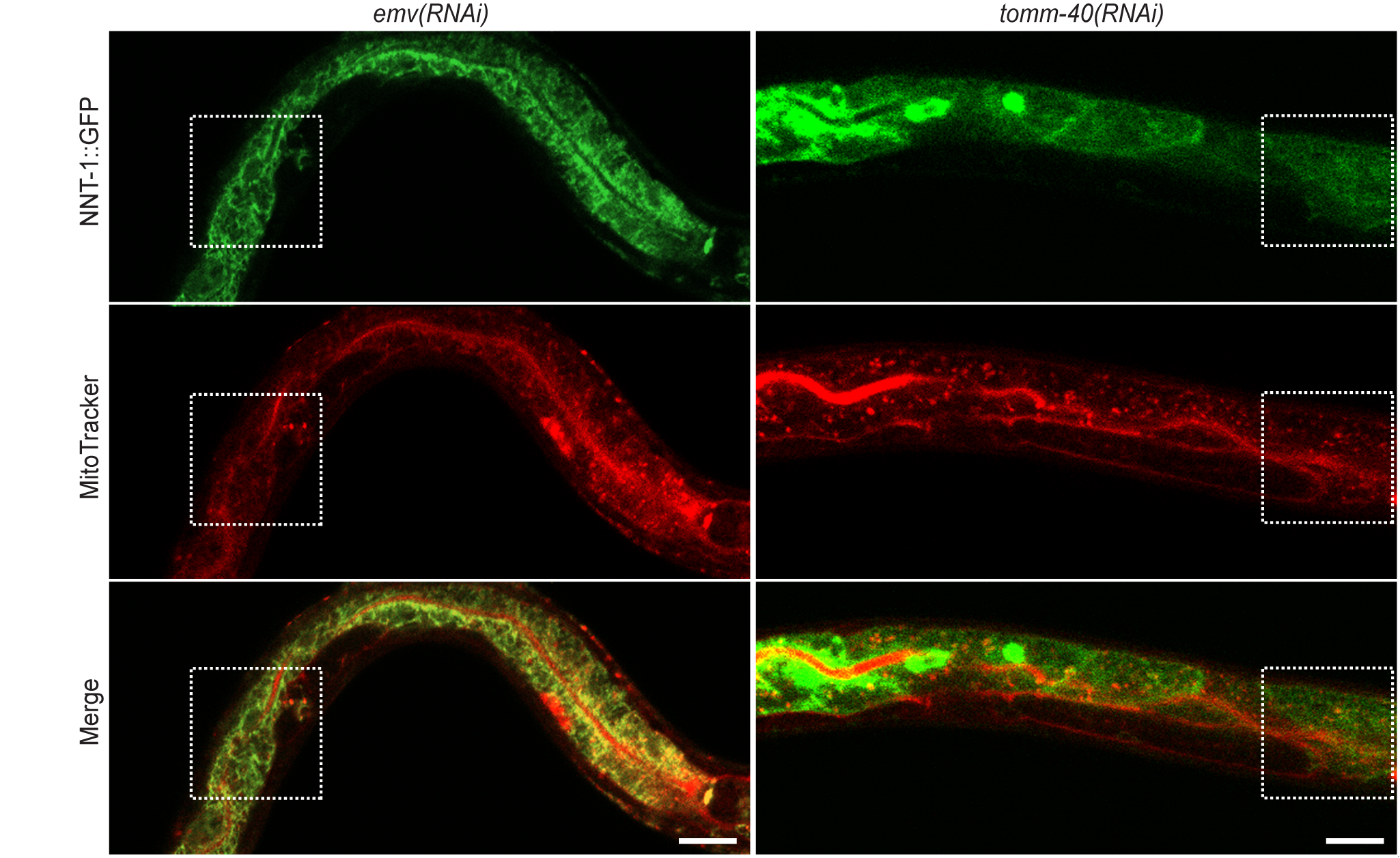

Supplement: Figure S2 — TOMM-40 is required for NNT-1::GFP to accumulate in mitochondria. Whole body confocal images of RNAi treated animals, transgenic for a truncated nnt-1::gfp construct and counterstained with MitoTracker. Dashed squares represent the portions that are enlarged in Figure 4A. Scale bars are 20 μm. (2.84 MB TIF) [file pone.0014507.s002.tif]

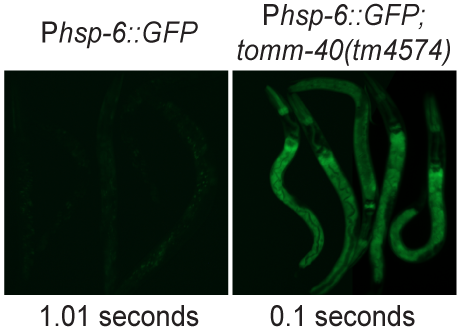

Supplement: Figure S3 — The mitochondrial stress response is evoked in tomm-40(tm4574) mutants. Fluorescence optics imaging of Phsp-6::gfp and Phsp-6::gfp; tomm-40(tm4574) animals. Images were captured at 1.01 seconds exposure time for Phsp-6::gfp animals and at 0.1 seconds for Phsp-6::gfp; tomm-40(tm4574) animals. (0.20 MB TIF) [file pone.0014507.s003.tif]

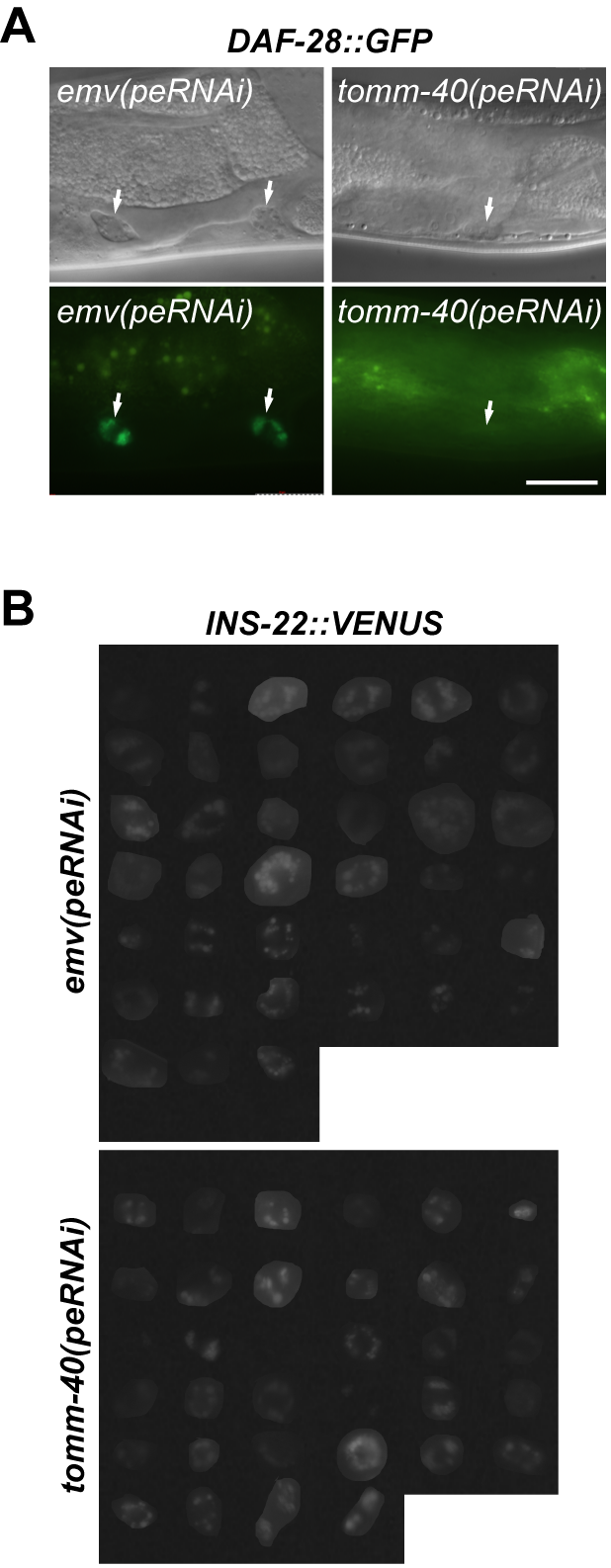

Supplement: Figure S4 — Accumulation of DAF-28::GFP and INS-22::Venus in coelomocytes. (A) Animals of the daf-28::gfp(svIs69) strain, treated with tomm-40(peRNAi) or emv(RNAi). Top panels are DIC representations of the fluorescence images below. (Left) An emv(peRNAi) animal, with vibrantly GFP-labeled coelomocytes. This animal was scored as secretion competent. (Right) A tomm-40(peRNAi) animal, with absence of any GFP-labeled coelomocyte. This animal was scored as secretion defective. Arrows indicate coelomocytes. The scale bar is 25 μm. (B) Picture representation of the coelomocytes that were measured for pixel intensity in Figure 6F. (Top) A panel showing 33 coelomocytes, from individual emv(peRNAi) worms, containing INS-22::VENUS and (below) 34 coelomocytes, from individual tomm-40(peRNAi) worms, containing INS-22::VENUS. (0.67 MB TIF) [file pone.0014507.s004.tif]
